# Exploring Well Water Testing Behaviour Through the Health Belief Model

**DOI:** 10.1177/1178630220910143

**Published:** 2020-03-11

**Authors:** Abraham Munene, Jocelyn Lockyer, Sylvia Checkley, David C. Hall

**Affiliations:** 1 Department of Ecosystem and Public Health, Faculty of Veterinary Medicine, University of Calgary, AB, Canada; 2 Department of Community Health Sciences, Cumming School of Medicine, University of Calgary, AB, Canada

**Keywords:** Health behaviour, intervention programme, interviews, risk perception, rural, water testing, well water

## Abstract

Health problems can arise from consuming contaminated well water. Well water testing can help prevent negative health outcomes associated with consuming contaminated water. The aim of this study was to understand the factors influencing well owner decisions to conduct water testing through the theoretical lens of the Health Belief Model. We conducted semi-structured interviews with 20 well owners and used framework analysis to sort and analyse the data. The results demonstrated that well owners’ perceived susceptibility to well water contamination was low, while the perceived severity of contamination, benefits of testing, and self-efficacy towards testing were high. Cues to action to promote testing focused on increasing well owner education and awareness through well stewardship programmes and reminders to test. Participants faced some barriers to water testing. Increasing education and awareness about well water contamination and water testing, while reducing logistical barriers to testing, may improve compliance with water testing.

Health problems could arise from drinking contaminated well water. Well water contaminants are associated with several illnesses including gastrointestinal illnesses, cancer, reproductive issues, and neurological disorders.^1^ Colorectal and lung cancers have been associated with the consumption of contaminated drinking water.^2,3^ Private water wells are an important domestic water source serving more than 10% of Canadians.^4,5^ Approximately 10% of Alberta’s rural residents rely on private wells for household use.^6^ Private wells in Canada may be susceptible to waterborne disease outbreaks.^4,7,8^ Microbiological pathogens in contaminated well water may cause gastrointestinal illnesses.^8,9^ Water wells may also be vulnerable to physiochemical contaminants such as arsenic, manganese, and fluoride.^10-12^ A recent study in Alberta found the prevalence of *Escherichia coli* and total coliforms in private wells to be 5% and 22%, respectively.^13^ Currently standards for drinking water in Canada state that there should be no *E. coli* or coliforms present in drinking water. Well contamination can occur through several scenarios that either impact the aquifer or the well. Weather or climatic conditions (eg, flooding), old, damaged or uncapped wells, proximity to potential contaminant sources such as manure, sewage, landfills, industrial activities, formation of biofilm in the well, and the natural geology of the aquifer are all factors that may be associated with well contamination.^14-17^

To protect well owners from the risks associated with consuming contaminated well water, the government of Canada recommends at least 2 microbiological tests per year and 1 chemical test every 2 years to assess for both microbiological and chemical contaminants. Well owners are responsible for testing their drinking water, deciding which tests to conduct (ie, microbiological or chemical) and how often they should conduct testing.^18^ Predicting the sources and timing of well contamination can be difficult because there are several factors that could influence the aquifer or well water quality.^19,20^ Well water testing becomes crucial in detecting well contamination and potentially protecting well owners from diseases associated with consuming contaminated drinking water.

## Microbiological Well Water Testing in Alberta

Well water testing services for microbiological contamination (ie, assessment for the presence of *E. coli* and total coliforms) as well as chemical testing for physiochemical contaminants is currently offered at no charge to well owners in Alberta through Alberta Health Services (AHS). Well water sample submissions are facilitated through more than 100 health centres across the province where well owners can pick up and drop off water sampling bottles. Due to the nature of the testing kit used, samples for microbiological analysis must be received at the laboratory within 24 hours of collection for the water sample to be viable for testing.^13,21^

A low proportion of well owners comply with testing recommendations set within their jurisdictions.^6,22,23^ Summers^6^ found that only about 10.7% of well owners in Alberta tested their water on an annual basis. Similarly, only about 20% of well water owners reported adhering to provincial testing recommendations.^22^ Some of the major barriers to well water testing identified in previous studies in Canada include inconvenience and time issues associated with well water test testing and a lack of information on well water testing.^22,24^ Microbiological well water testing recommendations vary. For example, while Alberta provincial recommendations are set at twice a year, federal guidelines may recommend testing up to 3 times a year while the World Health Organization recommends testing a well once it is drilled and as the situation demands on a regular basis.^18,25-27^

## Theoretical Model

Theories applied to well water stewardship include the Common-sense Model;^28^ the Risk, Attitudes, Norms, Abilities, and Self-regulation Model model;^23^ and the Health Belief Model (HBM).^29,30^ The HBM assumes that decisions to adopt a health behaviour are based on 6 constructs that influence perceptions of health risks.^31^ These constructs are perceived susceptibility, perceived severity, perceived barriers, perceived benefits, cues to action, and self-efficacy.^31,32^ Perceived susceptibility and severity make up threat perception related to the risk of well water contamination, while perceived barriers and benefits, cues to action and self-efficacy relate to the adoption of testing as a mitigation strategy to the risk.^29^

To help understand well water testing behaviour in the rural Alberta context, we conducted a qualitative study. Our aim was to explore and understand microbiological well water quality testing behaviour viewed through the theoretical lens of the HBM within the rural Alberta context. The HBM has been used to explain health behaviours, such as testing, screening for disease, and in the development of health promotion and intervention programmes for smoking, diagnostic exams, vaccine adoption, dieting, sexual risk-taking behaviour, exercise, and adoption of drinking water disinfection.^32-37^ To the best of our knowledge, only 1 study has assessed the suitability of the HBM in explaining the adoption of well testing as a health behaviour among New England residents to mitigate against the risks of well water contamination.^29^ Therefore by understanding well water testing behaviour using the HBM and how participants’ experiences with water testing align with the different components of the HBM, we can help understand the factors that act as barriers and facilitators to water testing within Alberta and tailor water testing programmes and well education to address these factors.

Qualitative studies are an important way of informing water policy and well water education programmes.^38-41^ Considering the low compliance with well water testing recommendations by well owners in Alberta^6^ and the inherent dangers of not testing, this study will be helpful in informing policy makers, regulators, educators, and environmental public health practitioners by helping them understand well owner perceptions about well water quality and the barriers that currently exist to well water testing. This information will be helpful in improving well water stewardship programmes and identifying ways to increase compliance with testing recommendations.

## Methods

### Study design

#### Participant recruitment

We primarily identified participants through the Alberta Well Water Information Database with requests to participate in the study sent through paper mail and email. Additional recruitment was done through online advertisements sent to watershed management groups, recruitment at well workshops, advertisements in rural newspapers, rural grocery stores, and community centres. We used a purposive sampling technique which involves selecting interviewees who are likely to generate useful and meaningful data to answer the research questions.^42,43^ Participants must have met the following criteria to be eligible for the interviews; participants had to be at least 18 years of age, must have had a water well which they used for domestic purposes (eg, drinking, washing, and cooking in the household), must have responded to the questionnaires sent previously. Furthermore, participants must have voluntarily submitted a recent microbiological well water quality test as part of the study before being interviewed.^41^ The study was conducted under the University of Calgary study number 1025400-3-1 and approved by the University of Calgary Conjoint Faculties Ethics Research Board and by the Research Ethics Board (REB13-0473). Consent to participate was obtained verbally during interview sessions.

### Data collection

We developed an interview guide to understand what factors were pertinent in influencing perceptions of well water quality in Alberta and what factors could be used to understand well water testing behaviour using the HBM. The study team consisted of 3 professors and the doctoral student. Interview sessions were conducted between May and August 2017. We conducted semistructured interviews either by phone or face to face at the participant’s home. All interviews were conducted in English. A preplanned questioning route was developed to increase consistency among the interviews and to increase the detail of responses given by respondents.^38^ We pretested the interview with 2 members of the study team and a participant to trial the preplanned questionnaire and probes. The duration of the interviews was between 20 and 40 minutes. Interviews were audio-taped and transcribed verbatim. Field notes were written during and after the interview sessions. All data were anonymized. None of the interviewers had any prior relationship with any of the participants other than initial contact to respond to the questionnaires.^41^ Saturation was determined when both code and meaning saturation were achieved, that is, no new themes arose from further interviews.^44^

### Data analysis

We used framework analysis to explore the subjective experiences of well water owners around the 6 constructs of the HBM.^45,46^ Framework analysis includes 7 steps: transcription, familiarization with the interviews, coding, developing an analytical framework, applying the analytical framework, charting data into the framework matrix, and finally interpreting the data.^45,46^ Interview transcripts were separately read and coded by 2 of the authors. Two members of the study team independently reviewed the data and met frequently to discuss concepts generated from the data in the process of familiarization with the interviews.

We developed major coding categories based on the constructs of the HBM through the questioning route. A codebook was developed by 2 of the authors and was reviewed by the study team. The generation of a coding system is important while conducting semistructured interviews as the codebook ensures that the concepts and ideas generated through the analysis are the same between coders enhancing the credibility of the findings.^47^ The codebook was created using qualitative data analysis software (NVivo 11 for Windows, QSR International, Melbourne, Australia).^48^ We selected the HBM as the analytical framework with the 6 constructs of the HBM being a priori themes. Subthemes were derived from the participants’ responses and discussions about the thematic categories based on the 6 constructs of the HBM. Excerpts from the interviews were used to populate the thematic categories based on the 6 constructs of the HBM with direct quotations from participants through charting and indexing. To interpret the data corpus and evaluate how well participants perceptions were appraised with the constructs of the HBM, 2 members of the study team (ie, the first 2 authors) independently ranked participants comments as either low, medium, or high in relation to 5 constructs (ie, susceptibility, severity, barriers, benefits, and self-efficacy) based on the wording and phrases participants used when responding to questions based on the constructs of the HBM (eg, if a participant stated ‘I do not think contamination is very likely’ when asked about susceptibility of contamination of their wells, their statement was ranked as low). The proportion of participants whose statements ranked as low, medium, or high were then tallied (see Table 2). We could not rank cues to action as this construct was based on participants proposed approaches to increase compliance with well water testing.

The foundations of rigour in qualitative research are based on the credibility, transferability, dependability, and the trustworthiness of the completed research.^47,49^ To ensure the credibility of our research, the research team had extensive discussions throughout the interviews and analysis. To increase transferability and dependability, the codebook used was reviewed by the study team, the transcripts were coded by more than one member, and agreement regarding coding was established through regular meetings.

## Results

### Participant characteristics

We interviewed 20 well owners. Participants were predominantly men (n = 13) who resided on farms (n = 14) and acreages in Alberta. The median age of participants was 58 (range: 35-74) years. All participants submitted a well water sample before being interviewed as part of this study. In addition to the sample submitted, well owners were asked about their well water testing habits, that is, whether they had previously tested, how often they tested their water, and the process of testing. Half of the well water owners interviewed stated they had conducted well water testing at the minimum recommended frequency of once per year or more. The prevalence of *E. coli* and total coliform contamination in our sample was 5% and 15%, respectively. Additional participant characteristics (eg, age, sex, income, and education) are reported by Munene et al.^41^

### Constructs of the HBM as they relate to well water testing

Water testing as framed within the context of the 6 constructs of the HBM and the subthemes informing decision making are presented in Table 1. Exemplary quotes are provided to reinforce the themes and subthemes.

**Table 1.** Summary of themes, subthemes, and quotes.

Constructs of HBM	Subthemes	Quotes
Susceptibility	Proximity to threat (eg, livestock and oil & gas)	‘you know . . . I don’t think there is a great risk of well contamination from the local activity . . . right at this point and time it is not a concern for me, due to our location and proximity of cattle grazing and the amount of land that is actually physically back there’.
	Current well stewardship practices (ie, treatment and testing)	‘. . . like keep it clean, chlorinate, check your stuff so you know it’.
	Current on farm/acreage practices	‘Um I do not think it is very likely because of the volume of the water, now its possible but not entirely likely . . . I am not worried about it because of the system we have on our water and the precautions we have taken’.
	Well infrastructure (ie, location of well and depth of well)	‘Uh . . . I would say by everything that we have done and the people before us by drilling the well where they did . . . I would say we have quite low risk of any well water contamination . . . I mean the well is in a location where I can look at it any time I want to make sure there is not a problem showing up . . . I would say quite low risk (of well water contamination) yeah’.
Severity	Illnesses and mortality associated with well water contamination	‘Well I think it can be pretty severe, mainly in terms of if there is a chemical contamination that could cause some health issues and bacteria could certainly cause some uh digestive kind of G.I. tract kind of stuff that could definitely make people very sick, and if they happened to be sick already you know, it could be dangerous . . .’
	Problems associated with finding alternative water sources on property in the event of contamination	‘Oh yeah it’s a real concern . . . If it’s a 1 to 6 scale, I would rate it to 6 as a concern. I look at the implications of what would happen if we did have a contaminated well, we’d have to move . . . or haul water in. When you see people hauling water in, what a disaster’.
	Implication on livestock health	‘We run livestock and some . . . certain levels of the different things that compose the water can affect the health of the livestock as well. So we have to keep that in mind always to be able to manage our livestock’.
Barriers	Delivery of well water samples to sample drop-off locations	‘No other than the fact that it is really inconvenient the times to test the well, if it would make it easier for people to just drop their samples . . . and I am not sure, you know the chemical analysis, whether it has to be within a time frame, but its really awkward to get that done . . . other than its very difficult because, if you are working you have to have the sample taken, two hours before you send it in, and in . . . I have to have it in by nine o clock’.
	Hours of operation of sample drop-off locations	‘Oh, do you know why they do not do it . . . There is a time window, so you go and you have to pay first of all, which I do not care, but some people have to pay ten dollars to get the bottle, and in our small towns you can only get it from the hospital, and you can only get it on certain days, so it is very restrictive. So, like for us we have to go to the . . . You can go Monday, Tuesday, Wednesday . . . that’s it. You have to be there between 8.00 in the morning and 5 in the afternoon, and you have to pay 10 bucks a bottle, and then you have to fill that bottle and then within that 24 hours have it back into them, so if you do not live near town, it becomes almost like you can’t do it. You have to take two days off, you’ve got to take one day to take the bottle, bring it home, fill it up, take the next day to run it in. And it’s got to be Monday, Tuesday or Wednesday’.
Benefits	Safeguard human and animal health	‘I think having them tested is going to tell us what is going on, with our water and therefore should be able to help us catch anything that might be coming in. Um . . . I think safety, knowing what is in your water and having it tested often enough tells you, you know where you are at, where your water is at and where you need to do the maintenance properly.’
	Give peace of mind over well water quality	‘Uh . . . its just peace of mind I guess, to know what our well is like.’
	Knowledge of the state of the aquifer well is tapping into	‘I think it is everybody, because if one of us was to find something really crazy in our well water, we would all be thinking holy (explicative), where is that coming from . . . what if its in an aquifer in the area.’
Self-efficacy	Well water testing is a relatively easy task to conduct	‘Easy enough . . . Yeah the results come back in a couple of weeks or three weeks and they are always precise and always good. Give me the information that I need.’
Cues to action	Home delivery service for sampling	‘Well I guess the only thing that would make it for me easier. Is if they could mail the samples or . . . have someone pick them up.’ ‘Once a year maybe even, like a mobile truck, or something that can have the right temperature, or however it is they need, like a mobile truck that says . . . ok I’m coming to this area at this time, you know, come and meet me or I can stop by or you know.’
	Education and awareness initiatives	‘I guess an awareness program, flyers in the mail box, you know if you have well water be aware of the dangers and be aware. You know something to make people aware, not scare them, but just a flyer, information sheets, fact sheets about wells and in general just to create more awareness would be a good thing.’
	Do it yourself home testing kits	‘I think the best thing is if they could have an at home kit (laugh). People could do it if they could do it in their own houses, they’d do it. Lots. The government should come up with an at home kit.’

#### Perceived susceptibility

Many participants (n = 14) stated that they did not feel they were susceptible to well water contamination or there was a low risk of contamination to their water wells. Participants commented that the mitigation strategies they used (eg, well treatments, and well maintenance and on property mitigation strategies) were enough to protect them against well water contamination. Participants also stated that the characteristics of the water wells such as where it was located, the well’s accessibility, and the depth of the well as reasons for their perceived lower susceptibility of contamination. For example, when asked about susceptibility to well water contamination one well owner said:

I think it is very unlikely . . . The depth of the well I guess, you know no cattle, no manure, in or around that area and that nothing can get in through the cap. It was formerly a well pit and I think it was at great risk for contamination because it had flooded in 2013 and so I had to have it changed and have that whole well system removed . . . I think it would be very difficult to do [contaminate] where it is located and where it is set up and managed now. . . . that really would almost be impossible for it to be contaminated.

Despite many participants (n = 14) expressing low perceived susceptibility towards well water contamination, a few participants (n = 5) felt that their wells were at risk of contamination. Some participants (n = 3) were unsure about the source, mechanism, and time at which well water contamination could occur; however, sentiments towards susceptibility to contamination were still low among these participants, and they considered the risk of their well getting contaminated as minimal. For example, when asked about how likely it was for them to have contaminated water well, one well owner mentioned ‘Things change, industries change. It (worrying about contamination) doesn’t keep me awake at night. If that makes any sense’.

#### Perceived severity

Most participants (n = 16) felt that the consequences of well water contamination on their health, their family’s health, the community, or livestock could be very severe. Sentiments expressed included very adverse consequences of well water contamination such as death, serious illness, and being unable to use water on their property in the event of well water contamination. For example, one well owner stated the implications of well water contamination could be dire and mentioned the Walkerton incident, one of the most notable water contamination events in Canada in which the contamination of a town well led to the deaths of 7 people with over 2000 becoming severely ill^50^ of ‘. . . I guess if you look at Walkerton it can kill people’. Nonetheless, some participants (n = 2) felt that well water contamination did not necessarily have to have severe consequences. For example, one well owner stated when referring to the level of microbiological contamination.

. . . but ok let’s say if your bacterial count must be zero (for the maximum acceptable concentration of *E. coli* and total coliforms). We’ve had it tested and it was literally 1 . . . just the coliforms, and they (the public health officials) are like yeah, your well is contaminated.

#### Perceived barriers

The inconvenience of dropping off a water sample coupled with limited hours of operation at drop-off health units were a constraint to the well water sample submission process. Well owners expressed (n = 7) that it was difficult to submit a sample especially if the hours of operation for the health centre conflicted with their schedules. A few participants (n = 5) expressed that they had to make special trips (ie, plan trips outside their routine) to submit samples.

Despite the constraints stated by participants in the logistics of submitting a well water test, most of the participants reported that the procedure was relatively ‘easy’ (n = 17). Furthermore, participants (n = 20) expressed satisfaction with the no charge well water testing programme currently being offered by the AHS. Feedback times for result reporting to participants were noted as timely for bacterial testing. However, participants did note that there was a longer wait time for result reporting when chemical tests were run on their well water. Participants also expressed trust in the testing process and the health officials conducting the tests. When asked about barriers, one well owner said ‘No (issues with testing). You just have to make a trip into town when they need it. I guess that’s the only barrier’. Another participant stated,

. . . I think it (testing) is very well done. Probably the only inconvenience is having to wait till 9 o clock to draw the water then to get it in.

Participants identified factors that they felt would help eliminate barriers and motivate well owners to conduct testing more frequently. Due to the inconvenience of picking up and dropping off water bottles at health service centres, having a delivery service for the water bottles, mailing the water bottles to the households, or having a home testing kit were raised as possible measures to reduce barriers to testing. One well owner suggested that having a ‘home testing kit’ may help eliminate barriers to testing. Another participant mentioned having a mail out service for water bottles:

. . . you know because that is limiting (hours of operation) that going to town between the hours of, I think ours are 9 am and 2 pm on a Tuesday or something. You know, if they offered a mail out service . . .

Although well water sample delivery services were discussed, some participants (n = 3) expressed that there were constraints of having such a service offered by the government. The additional costs of running such services and the possible reluctance of having public health officials going on private rural properties to collect water samples were raised as possible difficulties in implementing a water test delivery service. Some participants (n = 2) raised the issue of implementing mandatory well water testing regulations within the province. However, the logistics associated with the costs of enforcing such regulations was cited as a major barrier with enforcing the legislation. One well owner mentioned when discussing the costs of implementing a delivery service:

. . . I don’t think the taxpayers should pay anymore money for the government to come and get my (water) sample. I think as an owner; I am choosing to live on this land. I am choosing to have to learn and deal with well systems. It’s my responsibility to take care of myself, not the government.

When discussing the costs and logistics of a well water sample delivery service, another well owner stated,

Phone people, say you are from the government doing the testing for bacteria, take the sample. I think quite a few people would say, yeah that’s a good thing. But I also think there are quite a few people who are really hesitant just to have somebody come and test. Are you going to put anything in my water? And all that kind of stuff. Well then you gotta say feel free to bring in the sample yourself . . . maybe it’s a mix of both, but I can see the huge costs behind this.

An additional constraint mentioned by well owners was some difficulty in filling out the water testing requisition form. Two participants expressed that some of the information that is required to be filled on well water test requisition form may have been a bit difficult to obtain (ie, the geolocation in latitude and longitude coordinates of where their well was located). One participant mentioned,

If you know it (the testing service) is there, it is a great service (the free testing) and again it is easy, they provide nice bottles, the labelling identification is relatively easy . . . you have to work at the labels, have you seen the labels . . . and one thing they did ask for I think is the legal land description, uh it was a latitude and longitude. The GPS coordinate I can get easy, but the latitude and longitude. They wanted it in minutes and degrees.

#### Benefits to well water testing

Most well owners felt that there were benefits to well water testing (n = 17). Participants described the value of protecting their own and their family’s health. They noted that well water testing was a diagnostic measure that assured well owners that their well water was safe to use and that it gave them ‘peace of mind’ over their home water source. Participants also stated that water testing to monitor for any potential problems with the well was a ‘good’ thing to do. Health benefits were not only framed to human health but also to livestock using the same well water source. Ensuring that well water was safe to drink for livestock to maximize productivity was noted as important. For example, one well owner stated when discussing the health benefits to their livestock that ‘Well just to be sure there are no problems with anything, health wise you know . . . and you know because its for livestock as well, livestock health can be affected by bad well water too’.

Some participants (n = 3) noted that having their water tested for contaminants could also help them maintain the water infrastructure (eg, piping, toilets, and sinks) within their household. One participant mentioned when discussing benefits to water infrastructure within the home ‘. . . and even saving appliances with the hard water, if you can get that taken care of, your appliances will last longer, so better health and better lasting everything’.

Benefits of testing were also viewed in the context of the community with some well owners (n = 6) noting that recognizing problems with their wells may signal wider contamination of the aquifer within their community and therefore testing was a way of keeping themselves and their community members safe. For example, when discussing the benefits of well water testing, Participant 10 stated ‘Well it’s beneficial to the community because most of the people out here now have tested their water and they know that this region that we live in there is widespread nitrate contamination’.

#### Self-efficacy

Self-efficacy was determined by a well owner’s belief that they could conduct well water testing. All well owners (n = 20) expressed that conducting a well test was relatively easy, and all participants had been through the process of getting their microbiological well water tests done as part of the study and were therefore familiar with the process. Participants 1 and 15 stated,

I think if you are offering a free test it’s just as simple as picking up the bottles and dropping them back off again. I do not know what more incentive there can be. The incentive must come from the person too, you know. The test is free. Just get out and do it.

No (no issues with doing the testing). I just get sample bottles from the health unit here and I take samples and I give it back to them and they send it to the laboratory and it gets tested and they come back with the results within a few days and that’s it, and I do that once a year.

#### Cues to action

Well owners recognized the need for creating more awareness of the well water testing programme and recommendations in the province. Educational initiatives and advertisements through local media were proposed as ways of raising awareness towards well water testing. Participants 4 and 11 stated,

I know a lot of people that number one: They do not know it is free, they do not know the facility exists, they do not know how to do it. So, some more . . . advertising or I guess education from the agency to the public would certainly be helpful and I guess certainly some education to suggest that the need is important. Do not just do it because it is free, and it is there. Do it because you should do it.

. . . more public information, because you got all kinds of people moving out into rural areas and they haven’t got a clue as to where their water comes from. They haven’t got a clue that they need it to be tested and I only found out that I should be shocking my well every year, and I’ve been here for 8 years, you know, and so there you go. Proof is in the pudding that homeowners and landowners need to have their wells tested.

### A synthesis of the HBM and water testing behaviour

The alignment of participants’ statements with the constructs of the HBM, how participants ranked them, the subthemes discussed within the context of 5 constructs of the HBM is presented in Table 2. Proximity to threat, current stewardship practices, and well infrastructure seemed to play a role in the participants’ evaluation of susceptibility to well contamination. Delivery of well water samples, hours of operation of water bottle centres, lack of awareness of testing services, forgetfulness, or procrastination were the main barriers to testing. Illnesses associated with well water contamination, issues with finding alternative drinking water sources, and implications on livestock health and productivity were the main issues raised when discussing severity of contamination. Benefits of testing included protecting human and animal health, providing peace of mind over well water quality, and providing knowledge of the state of the aquifer. With respect to self-efficacy, well owners acknowledged that microbiological testing was fairly easy to conduct with the major barriers being logistical in delivering water samples.

**Table 2.** Alignment of participants’ statements with the HBM.

Rank	Construct of HBM	Subthemes	Number of participants whose statements aligned with the rank
Low	Susceptibility	Proximity to threat	14/20
		Current well stewardship practices (ie, treatment and testing)	
		Current on farm/acreage practices (eg, fencing off well area and limiting land use around well head)	
		Well infrastructure (ie, location of well and depth of well)	
Low-medium	Barriers	Delivery of well water samples to sample drop-off locations	17/20
		Hours of operation of sample drop-off locations	
		Lack of awareness of no charge water testing services	
		Reminders to conduct water testing on a more frequent basis	
High	Severity	Illnesses and mortality associated with well water contamination	16/20
		Problems and expense associated with finding alternative water sources on property in the event of contamination	
		Implications on livestock health	
High	Benefits	Safeguard human and animal health	18/20
		Give peace of mind over well water quality	
		Knowledge of the state of the aquifer well is tapping into	
High	Self-efficacy	Well water testing is a relatively easy task to conduct	20/20
		All well owners tested their well water quality as part of the study	
		Few barriers were mentioned in terms of the process of conducting a water test	

Abbreviation: HBM, Health Belief Model.

For the group interviewed, perceptions of susceptibility to water contamination were low. The severity of well water contamination was framed in terms of the consequences to human health (ie, illness and mortality). Furthermore, consequences were also framed in terms of the problems of getting alternative water sources on the property in the event of contamination, maintaining appliances, and production loss because of well water contamination if well water was used for livestock. While all owners had tested their water as part of the study, a few barriers were noted in the submission process including the logistics of getting a sample in for water testing, the hours of operation of the sample drop-off health facilities. Elimination of barriers faced by well water owners testing (ie, logistical issues in submission of samples) were noted as some of the major ways to remove barriers towards well water testing in the province.

Benefits accrued from well water testing included safeguarding human, animal, and community health. Having knowledge of well water quality and ‘peace of mind’ over the drinking water sources were also noted as important to well water owners. As all well water owners had tested their water for this study, many well water owners believed and found the process of well water testing as feasible. Cues to action were presented as ways that would motivate well water testing behaviour. Strategies suggested increasing well owner awareness, education, increasing the visibility of the free well water testing service, and reminding well owners to test. Engaging in the study was also a cue to action for well owners who chose to submit water samples.

## Discussion

The aim of this study was to explore and understand microbiological well water quality testing behaviour viewed through the theoretical lens of the HBM. The framework analysis using the HBM delineated individual-level perceptions and constraints faced by well water owners in conducting testing. The decision to conduct well water testing was based on people’s perceptions of their susceptibility to contamination, the severity of the consequences of contamination, the benefits of well water testing and the barriers they faced to getting it done. Decision making was also influenced by the cues to action they received and their self-efficacy. They believed there were cues that might help people achieve higher rates of compliance.

Well owners were confident in the safety of the drinking water from their wells and felt that their wells were not susceptible to contamination. Similar findings have been reported among well owners in Canada.^6,38,40^ This is congruent with previous studies assessing the suitability of the HBM where susceptibility was found to be a weak predictor of the adoption of a health behaviour.^29,51^

In addition to severe negative health outcomes such as mortality and morbidity, the severity of well water contamination was also framed as severe because well owners would need to find and purchase alternative water sources. Worrying about the potentially negative health impacts of well water contamination has been found to be an important factor in decisions to conduct well stewardship practices such as testing, treatment, or in the choice to have alternative drinking water sources.^38,52^ Well water contamination can have a range of health consequences depending on what contaminants individuals are exposed to, the level contaminants individuals are exposed to, the duration of exposure, and the hosts’ immune system. For example, while microbiological well contamination by *E. coli* may lead to gastrointestinal illness in healthy adults, it may have more severe consequences in very young children, the elderly, and immunocompromised individuals.^9^

Well water testing mainly served to bring peace of mind to participants and provide confidence in the safety of their drinking water. Providing peace of mind to well owners about the safety of their well water quality has been identified as an important benefit of well water testing.^6,38^ Identifying that some well owners may perceive additional benefits beyond their own personal or family health (eg, to their livestock or communities) may be an important communication message to encourage testing because it recognizes and understands the diverse experiences and perspectives among well owners.^53^

Participants identified logistical constraints in submitting water samples. Other studies have found the inconvenience of submitting well water samples for testing as a barrier.^22,38^ However, as well water owners had conducted water testing as part of our study, some of the perceived barriers towards well water testing may have been ranked lower. Hexemer et al^54^ reports similar findings where some of the perceived inconveniences of water testing were removed from their study (ie, getting well owners to go through the process of testing and delivering testing kits).

All well owners were able to conduct the test and most of the well owners in this study acknowledged that conducting a well water test was feasible. The high self-efficacy shown by the participants in conducting well water tests was a positive indicator that with the current AHS programme, conducting well water testing was feasible.

Cues to action were factors that would motivate well water testing behaviour. Factors that motivate well water testing are useful because they could be targeted by health officials to increase compliance with water testing.^29,38^ Most recommendations stated by well water owners to increase compliance with testing had to do with educating well owners about the importance of well water testing and stewardship, raising awareness of water testing services through media, and reminding well owners to conduct testing. Factors such as reminding well owners to conduct testing and providing minor incentive for well owners to conduct testing were cited as possible cues to action in reminding well water owners to conduct well water testing in the future.^29^ Increasing well owner education about well water stewardship practices and the importance of well water testing is currently facilitated by the Working Well Programme (WWP) in the province. Increasing the visibility of this programme and providing more access to well water stewardship information through media may help increase compliance with testing.

Our findings have important implications for the delivery of well water services. Most well owners perceived their well water as safe. Educational messaging could focus on improving environmental public health literacy enabling well owners understand potential hazards, sources of contamination, mechanisms that could lead to well contamination. As well owners identified logistical problems in getting their water tested, these concerns may be mitigated by finding ways to make the submission of samples easier to increase compliance.^53^ Increasing the visibility of the no charge well water testing service and well owner education programmes such as the WWP may be an important step in raising awareness about the services currently offered. Having community-driven initiatives based on public health engagement with watershed management and local community groups may be an important strategy in increasing well owner education and compliance with well stewardship practices.^52,55^

### Limitations

There are limitations to the study. The findings are situated within a narrow profile of well owners in Alberta with a specific set of potential risk factors to well water contamination found in Alberta at a specific time and, therefore, may not be transferable to other settings. The presence of the no charge well water testing programme, well water stewardship initiatives such as the WWP, and contextual factors unique to Alberta^41^ may make well water testing behaviour nontransferable to other jurisdictions. We used a HBM-based deductive approach to understand well water testing behaviour which may have biased other potential explanations for the behaviour. As participants in our study had conducted a well water test as part of the study before being interviewed, this could have potentially influenced their perceived susceptibility to well water contamination.^33,51^ Furthermore, as we only had information on participants who conducted testing, we had no information why other well owners who were requested to submit water samples did not test. Barriers to testing, education, and income were found to influence past well water testing behaviour.^29^ However, although we found barriers to well water testing specific to the Alberta context, we were not able to assess for the role of modifying variables such as education and income on well water testing behaviour. This may require future research conducted with a quantitative design.

## Conclusions

We carried out an in-depth study to explore and understand factors influencing well water owner’s decisions to conduct water quality testing as viewed through the theoretical lens of the HBM. To the best of our knowledge this is the first qualitative paper to examine how the HBM can be used to understand well water testing behaviour. The HBM was useful in understanding individual behavioural factors that influenced well water testing. We determined susceptibility to well water contamination to be low among our participants while most participants appraised the severity of well water contamination as high. Although most well owners acknowledge testing was easy to conduct, believed they could conduct testing, and recognized the benefits of well water testing, there were logistical barriers in the submission of water quality test and most of the cues to action had to with increasing awareness and visibility of the well testing programme.

## Supplemental Material

Interview_guide_and_statement_ranking_409aa81a26_xyz266599a5f31e8 – Supplemental material for Exploring Well Water Testing Behaviour Through the Health Belief Model Click here for additional data file. Supplemental material, Interview_guide_and_statement_ranking_409aa81a26_xyz266599a5f31e8 for Exploring Well Water Testing Behaviour Through the Health Belief Model by Abraham Munene, Jocelyn Lockyer, Sylvia Checkley and David C. Hall in Environmental Health Insights
