# Supplementary material for: Exploring Well Water Testing Behaviour Through the Health Belief Model
Source: Environ Health Insights. 2020 Mar 11;14:1178630220910143. doi: 10.1177/1178630220910143 (PMC7077933; doi:10.1177/1178630220910143)
Supplement: Interview_guide_and_statement_ranking_409aa81a26_xyz266599a5f31e8 – Supplemental material for Exploring Well Water Testing Behaviour Through the Health Belief Model [file Interview_guide_and_statement_ranking_409aa81a26_xyz266599a5f31e8.pdf]

Interview questions.

Question 1: Tell me about the sources of the drinking water in your home?

Probe: How often do you drink water from your in-house tap?

Probe: Tell me about your satisfaction with the look, taste, odour, feel of the water you primarily drink in your homes?

Probe: How would you compare the look, taste, odour with other sources of drinking water you may have experienced (e.g. bottled water)?

Question 2: What comes to your mind when you hear of well water pollution?

Probe: Do you feel well water pollution is a risk in your area?

Probe: Do you think well water pollution is a priority issue with respect to health for example do you think of well water pollution like you would of heart disease or cancer?

Probe: What illnesses do you think can be caused by well water pollution?

Question 3: Tell me about the current well water management practices that you use to protect your water well from pollution.

Probe: Describe what led you to use these management practices (testing, treatment and other).

Probe: Tell me about your beliefs towards these management practices and whether you believe they protect you against illness.

Probe: Can you tell me how you test water in your home? How often are you able to test your well water in your home?

Question 4: I would like to talk a bit about livestock manure and how it might affect your well water. Do you have any thoughts about that?

Probe: Can you describe any experiences that you may have had or know about with respect to water contamination by livestock manure.

Probe: What livestock species are likely to cause pollution in your area?

Probe: Is there anything people can do to prevent well water pollution by livestock manure?

Probe: How feasible is it to prevent well water pollution by livestock manure in your opinion?

Probe: How would you rank livestock as a risk to water contamination relative to other potential sources of well water pollution in your area?

Question 5: How likely is your well to get polluted? Or Is your well likely to get polluted?

Probe: What scenarios do you think would increase the likelihood of your well being polluted?

Question 6: How dangerous (or severe) do you think well water pollution can be? Or How big of a problem do you think well water pollution is?

Probe: Do you know of any well water pollution events in Alberta (Canada)?

Probe: Might you be concerned about your family's health from water pollution by livestock?

Question 7: Can we talk about testing and treating your water and how you go about doing it

*Probe***:** Have there been any problems in getting your water tested? What were they?

Probe: Have there been any problems in getting your well water treated (and what were they)?

Probe: How might these problems be addressed (or what do you think are the possible solutions to these problems)?

Question 8: What would help you get your water tested on a regular basis?

Probe: Is there anything the well water testers or government can do to get you to test your well water?

Question 9: How might you and your family benefit from getting your water tested?

Probe: Might this benefit only your family or others? May you expand on this idea?

Summary table of +/-/n comments related to HBM as rated by the authors

Participant	Susceptibility	Severity	Barriers	Benefits	Cues to action	Self efficacy
1	+/n	+	-	+	N/A	+
2	-	+	n	+/n	N/A	+
3	-	n	-	+	N/A	+
4	+	+	-/n	+	N/A	+
5	+	+	-/n	+	N/A	+
6	-	+	-/n	+	N/A	+
7	-	-	+	+	N/A	+
8	-	+	-/n	+	N/A	+
9	-	+	-/n	+	N/A	+
10	-	+	-	+	N/A	+
11	-	+	n	+	N/A	+
12	+	+	-	+	N/A	+
13	-	+	-	+	N/A	+
14	-	+	-/n	+	N/A	+
15	N/A	n	-/n	+	N/A	+
16	-	+	-	+	N/A	+
17	-	+	-	n	N/A	+
18	-	+	-	+	N/A	+
19	+	+	+	+	N/A	+
20	-	n	-	+	N/A	+

Notes: + indicates high, - indicates low, n indicates medium
